# Atypical Presentation of Descending Aortic Dissection in an Acute Heart Failure Patient

**DOI:** 10.7759/cureus.17414

**Published:** 2021-08-24

**Authors:** May T Breitling, Swann Tin, William Lim, Abhiram Nagaraj, Richard Grodman

**Affiliations:** 1 Internal Medicine, Richmond University Medical Center, Staten Island, USA; 2 Radiation, Richmond University Medical Center, Staten Island, USA; 3 Cardiology, Richmond University Medical Center, Staten Island, USA

**Keywords:** atypical presentation of aortic dissection, systolic heart failure, atypical chest pain, debakey classification, stanford type a dissection

## Abstract

Aortic dissection is relatively uncommon, but often presents with acute severe chest or back pain and acute hemodynamic compromise and is associated with high mortality. We present a case of aortic dissection with an atypical presentation in a heart failure patient and the challenges encountered to make the diagnosis. The patient was a 54-year-old African American female who presented with progressively worsening exertional dyspnea and orthopnea for three days and sensation of indigestion and bloating. The patient denied any recent history of chest pain and she was initially admitted for heart failure exacerbation. Her admission chest x-ray showed severe cardiomegaly with a prominence of pulmonary vascular but there was a borderline widening of mediastinum measuring 8.2 cm. Physical exam showed unequal dorsalis pedis pulses (fainter on the right side) and systolic blood pressure difference of more than 20 mmHg between bilateral upper extremities. Computed tomography angiography (CTA) of chest, abdomen, and pelvis confirmed the diagnosis of dissection of thoracic and abdominal aorta extending from the left subclavian artery to the femoral artery. The patient was managed with labetalol drip and later transferred to a tertiary center for an elevated level of care where the endovascular intervention was performed. The patient then followed up with a vascular clinic for serial CTA and heart failure clinic for optimization of core measures. In conclusion, this case highlights the importance of clinical suspicion of aortic dissection and discusses the various clinical presentations of aortic dissection and its management. Being a highly fatal condition, prompt diagnosis is extremely important and is often life-saving. Therefore, it is important for physicians to be aware of atypical presentations of aortic dissection to initiate timely interventions to avoid catastrophic complications.

## Introduction

Aortic dissection is relatively uncommon and can be life-threatening if it is not diagnosed and managed promptly. The incidence of acute aortic dissection in the general population is estimated to range from 2.6 to 3.5 per 100,000 person-years [1,2]. Early diagnosis is the key, however, the diagnosis can be challenging sometimes since it can mimic other conditions such as acute coronary syndrome, pulmonary embolism, heart failure, or acute abdominal illness, leading to a risk of misdiagnosis [3]. About 14-16% of cases of aortic dissection presenting to the emergency department (ED) had an initial misdiagnosis [3,4].

## Case presentation

A 54-year-old female with a past medical history of hypertension, heart failure with reduced ejection fraction (HFrEF, EF <35%), left ventricular thrombus (not compliant with warfarin), obesity, hypercholesterolemia, chronic obstructive pulmonary disease (COPD), schizophrenia presented to the emergency room with a chief complaint of shortness of breath and a sensation of indigestion for three days. Shortness of breath was intermittent, aggravated by moderate exertion or lying down and relieved by rest or sitting upright. The patient has had a five-pillow orthopnea for many years and it worsened over the past three days prompting her to the emergency department (ED). Upon review of systems, the patient claimed that she had a bloating sensation with mild epigastric pain which radiated to the back intermittently and was relieved after one episode of vomiting. Numbness in bilateral lower extremities was present which has been ongoing for several months. The patient denied fever, cough, dizziness, syncope, numbness in the upper extremities. The patient claimed that she was not compliant with her medications as well.

**Figure 1:**
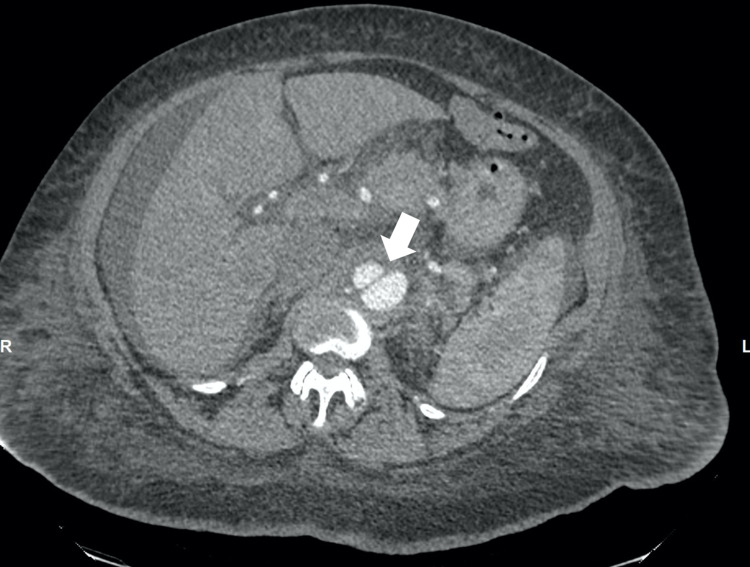
Computed Tomography Angiogram (abdomen and pelvis). The white arrow shows dissection at descending aorta.

**Figure 2:**
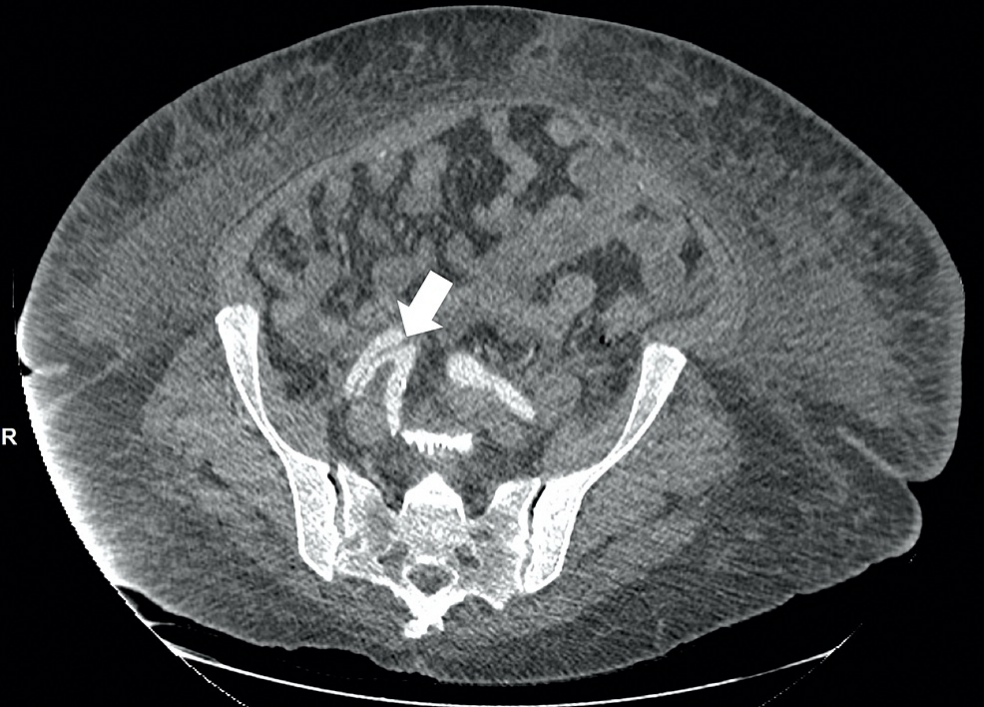
Computed Tomography Angiogram (abdomen and pelvis). The white arrow shows dissection extending into the right external iliac artery.

**Figure 3:**
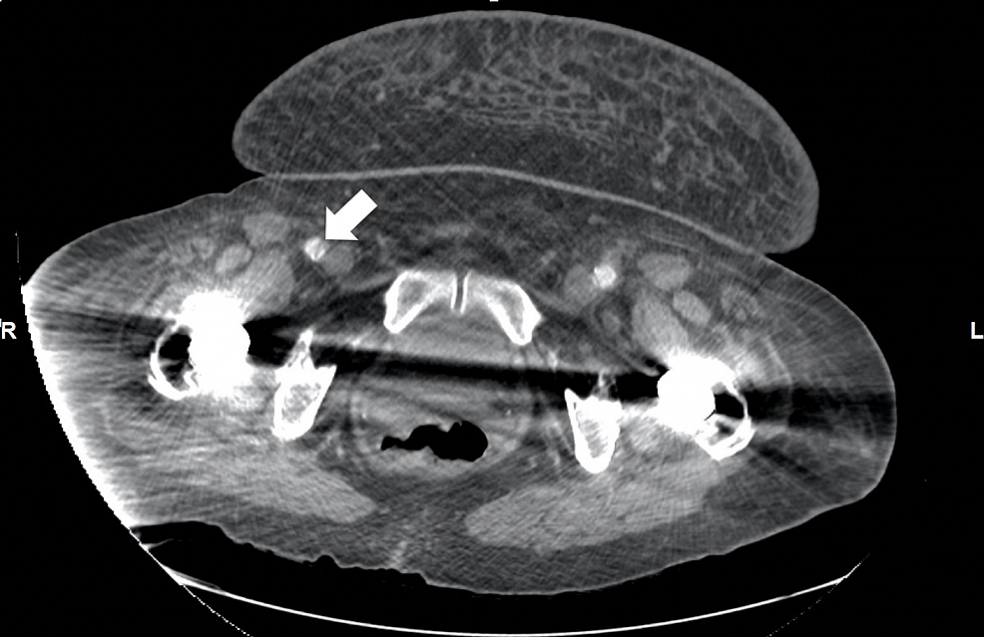
Computed Tomography Angiogram (abdomen and pelvis). The white arrow shows dissection extending into right femoral artery.

Upon examination, the patient was in mild distress due to tachypnea at 25 breaths per minute and hypoxic requiring supplemental oxygen (oxygen saturation of 95% on 4 liters of nasal cannula), pulse rate at 90 bpm, and regular and equal on both hands. Blood pressure measured automatically on the left arm was 195/129 mmHg and right arm was 163/90 mmHg and blood pressure measured manually on the left arm was 180/100 mmHg and the right arm was 170/110 mmHg. Cardiovascular examination revealed jugular venous distension, intact bilateral carotid pulses, normally first and second heart sounds with no gallop, rub or murmur. Pulmonary examination showed wheezing all over the bilateral lung fields along with bilateral basilar crackles, therefore, with signs of fluid overload. Lower extremities exam showed bilateral mild pedal edema with unequal dorsalis pedis pulsation (less on right but intact on left lower extremities). Initial chest x-ray showed severe cardiomegaly with prominent pulmonary vasculature. There was suspicion of borderline widening of the mediastinum measuring 8.2 cm. The basic metabolic panel showed acute renal failure with a blood urea nitrogen (BUN) of 42mg/dl and Creatinine of 1.7mg/dl. Due to a history of vague epigastric pain, unequal distal peripheral pulses in lower extremities, along with unequal blood pressure on upper extremities as well as suspicion of mediastinum widening, a computed tomography angiogram (CTA) of chest, abdomen, and pelvis was performed. It showed dissecting aneurysms of the thoracic and abdominal aorta (Figure 1) beginning just distal to the origin of the left subclavian artery measuring up to 4.7 cm in diameter, with extension into the right common iliac, external iliac (Figure 2), and common femoral arteries (Figure 3).

Labetalol drip was started for tight blood pressure control with goal systolic blood pressure of less than 110 mmHg and heart rate less than 60 bpm. The patient was admitted to the coronary care unit (CCU) for close monitoring. The patient was transferred to the tertiary center for an escalated level of care and surgical intervention.

Blood pressure was tightly controlled over seven days of admission to surgical ICU and endovascular intervention was later performed with TEVAR (Thoracic Endovascular Aortic Repair) and stenting in the thoracic and suprarenal abdominal aorta. The patient was discharged with a plan of following up in a vascular clinic for serial imaging follow-up with CTA at three, six, 12 months, and annually thereafter and heart failure clinic for optimization of core measures and follow-up for blood pressure control.

## Discussion

Aortic dissection frequently presents with sudden onset of severe chest pain with radiation to the back and acute hemodynamic compromise. The two most commonly used classifications for acute aortic dissection include the Stanford and DeBakey systems. The Stanford system categorizes dissections into those involving the ascending aorta (and may also involve the arch or descending aorta) as type A, regardless of the site of the primary intimal tear, and all others as type B [5]. The DeBakey system focuses mainly on the site of origin of dissection, with type 1 originating in the ascending aorta and propagating to at least the aortic arch, type 2 originating in and confined to the ascending aorta, and type 3 originating in the descending aorta and extending distally or proximally, but not proximal the left subclavian artery [6]. Chest pain is the most common presentation in aortic dissection which accounts for 79 percent in type A and 63 percent in type B dissections [7]. The pain is usually noted as a tearing or stabbing sensation and the location of pain usually correlates with the location of the dissection [8]. Painless dissection has also been reported in roughly 6.4% to 17% of cases [9]. When left untreated, about 33% of patients die within the first 24 hours, and 50% die within 48 hours [10]. 

Our patient initially presented with indigestion, bloating sensation, and mild abdominal discomfort. Atypical pain/painless dissection could sometimes be seen in diabetic patients, however, the patient doesn't have any history of diabetes mellitus. Also in acute heart failure exacerbation, abdominal discomfort is commonly interpreted as a sign of vascular congestion of the abdominal viscera. In our case, important cardiovascular exam findings such as differences in systolic blood pressure measurements and faint femoral and dorsalis pedis pulses led us to the timely diagnosis of descending aortic dissection. Therefore, this case report not only emphasizes the importance of performing a complete cardiovascular examination but also highlights the clinicians to be aware of both usual and unusual presentations of aortic dissection.

## Conclusions

This case report highlights the importance of suspicion of aortic dissection in heart failure patients since they can be presented with unusual symptoms. Aortic dissection is a highly fatal condition and both early diagnosis and timely intervention are crucial for life-saving. Even though it is relatively uncommon, it is important for physicians to be aware of atypical presentations of aortic dissection and to initiate timely interventions to avoid catastrophic complications.
